# Awareness of female malignancies among women and their partners in Southern Sri Lanka and implications for screening: a cross sectional study

**DOI:** 10.1186/s12889-015-2531-6

**Published:** 2015-11-25

**Authors:** Chamindri Witharana, Prabhavi Wijesiriwardhana, Kalani Jayasekara, Priyanka Kumari, Chaturaka Rodrigo

**Affiliations:** Allied Health Sciences, Faculty of Medicine, University of Ruhuna, Galle, Sri Lanka; Department of Clinical Medicine, Faculty of Medicine, University of Colombo, 25, Kynsey Road, Colombo 08, Sri Lanka

**Keywords:** Breast cancer, Cervical cancer, Uterine cancer, Sri Lanka, Mammography, Pap Smear

## Abstract

**Background:**

The incidences of breast, cervical and uterine malignancies continue to increase in Sri Lanka. It is important to explore the awareness of both women and their male partners regarding these malignancies and available screening services as it would determine the health seeking behaviours of females.

**Methods:**

This was a cross sectional survey of couples residing in the Galle District of the Southern province of Sri Lanka. The sample was selected from all 17 health administrative divisions of the district. An interviewer administered questionnaire was used to collect data on demography and level of awareness (risk factors, symptoms, signs, screening services) of breast, cervical and uterine cancers. Same questionnaire was used for both sexes except for gender specific questions.

**Results:**

A total of 282 (n-282, 564 individuals) couples were interviewed. The level of awareness regarding all malignancies was low. More than 50 % of participants in both sexes scored less than half the points on a questionnaire testing awareness. Better family income, better education and permanent employment showed a significant association with better awareness in both sexes (univariate analysis). Encouragement by male partner was associated with better participation in some instances.

**Conclusions:**

Community based health education on female malignancies needs to target both sexes. Educating males is important as, i) male partners can encourage females to utilize screening services and ii) some screening and preventive measures are relevant to males also. Better awareness of males may increase the uptake of screening services by females in societies with male dominant gender roles.

## Background

The overall cancer incidence in females in Sri Lanka shows a gradual rise after age 30, reaching a peak in the age group of 50–60 years [1]. Breast and cervical malignancies are two of the most common malignancies affecting women of reproductive and postmenopausal age groups and have crude incidence rates of 19 and 7.3 per 100,000 of population, respectively [1, 2]. Ovarian and uterine malignancies (e.g., endometrial carcinoma) are placed fifth and ninth in the list of common female malignancies [1].

The treatment costs of female reproductive system malignancies (including breast cancer) is a considerable burden to government healthcare expenditure and premature deaths of females are an irreplaceable loss to their families and workforce. Recognizing the importance of early detection and treatment of female reproductive tract cancers, successive governments in Sri Lanka have initiated and funded community based health education and early detection/screening programmes mainly through “well woman clinics” [3]. While the positive impact of these interventions are obvious (early detection via standard screening measures such as Papanicolaou (Pap) smear for cervical cancer), the numbers of female reproductive system malignancies in Sri Lanka are still significant and increasing [4].

Against this background it is important to assess secondary factors that may influence the health seeking behaviours of females with respect to screening and early detection. One significant yet, unassessed area in this regard is the knowledge, attitudes and practices of males regarding female reproductive tract malignancies. We hypothesize that given the traditional male dominated gender norms in Sri Lankan society, better awareness by male partners will have a significant positive impact on health seeking behaviours of females (e.g., attending voluntary screening). We also hypothesize that other confounding factors such as level of education of partners, family income, permanent employment, having a relative affected by cancer will also be associated with better awareness and positive health seeking behaviours of females. It has been shown previously that better education, age greater than 50 years, past history of hysterectomy and several other factors to be significantly associated with attending breast cancer screening in Sri Lanka [5]. However, this sample was selected from women attending a mammography centre and is not representative of the community. Another clinic based study demonstrated very low uptake for Pap smear screening among eligible women in Sri Lanka [4]. The reasons for this observation including that of gender roles and partner attitudes have not been evaluated so far.

The objectives of this study were a) to assess the awareness of symptoms, risk factors and available screening facilities for three common female malignancies, namely; breast, cervical and uterine malignancies among women and their male partners, b) to assess the percentage utilization of screening services and self-examination strategies for early detection by women, c) to correlate utilization of health services by women with awareness, level of education and acceptance of screening by male partner, and d) to correlate utilization of health services by women with level of education, family income and employment status of women.

## Methods

### Study design and sample selection

This was a community based cross sectional survey of married partners in the Galle administrative district of the Southern province of Sri Lanka. This region contains approximately 5.3 % of the population of Sri Lanka. Apart from areas surrounding Galle, the administrative capital of the province, other areas of the district are predominantly rural. Data on awareness of female malignancies in Sri Lanka from previous studies were mainly from Colombo in Western Province which is a metropolitan urban area. The Galle district is further divided in to 17 health administrative divisions termed Medical Officer of Health (MOH) areas. It was difficult to estimate the total number of married couples currently living in the district as this data was not available. All newly married couples within the MOH divisions have to be registered in an “eligible couples register” to be targeted for health education and service delivery with regard to contraception, pregnancy and family planning. We intended to use this register to approximate the number of married couples residing in each MOH division but the data was inadequate. From the available data from four MOH divisions (approximately 25,000 registered couples) we estimated the total number of married couples currently living within the district to be around 100,000. The calculated sample size at confidence interval of 95 % was 266 couples. The number was equally divided between each of the 17 MOH divisions. The selection of couples was random within each MOH division and continued on a house to house basis until the required number of samples per MOH area was interviewed.

### Data collection

The data collection was with an interviewer administered questionnaire that collected data on a) basic demography, b) awareness of male and female malignancies and c) utilization of screening and preventive services offered free of charge by the government (except mammography). The same questionnaire was used to interview males with exclusion of some questions specific to females (utilization of services such as well woman clinics). In addition, males were asked whether they had actively encouraged women to attend screening services and if they have had any objections on utilization of such screening services. The exclusion criteria were; non-consenting partners (male or female), dementia or concurrent illness interfering with effective communication (for both sexes), women with a past history of any of the malignancies studied and women less than 21 years of age and their partners.

The level of awareness was assessed on two domains (risk factors and symptoms/ signs) for all three cancers studied; breast, cervical and uterine (six sections of questions). Of the uterine malignancies focus was mainly on endometrial carcinoma. Each section consisted of 6–10 response items that had to be selected as a risk factor or a sign/symptom of the particular malignancy. Not all responses were correct. Each correctly identified response was given one mark under each section. Wrong answers were not given negative marks but they were used to identify non-specific answering (respondents answering “yes” to all questions). The marks for each subsection were calculated as a percentage and a cut off 50 % was arbitrarily accepted to define a satisfactory level of knowledge for each section.

The utilization of screening services were assessed in four aspects; self-breast examination (SBE), Pap smear to screen for cervical intraepithelial neoplasia/dysplasia, mammography, and attendance at well woman clinics. Well woman clinics are a free state-sponsored service available to all women aged 45 or more within their MOH division. In the clinics, women are educated on post-menopausal health problems including malignancies. Some screening services such as Pap smear are available within the clinics and for others eligible candidates are referred to hospitals. When calculating summary statistics, only eligible women were considered as the denominator for each screening method (age over 45 for attending well woman clinics, age over 21 for Pap smear and age over 40 for mammography) [6, 7].

The awareness of malignancies (in both sexes) and uptake of screening services (in females) were compared for significant associations with factors such as level of education, permanent employment, having a family income of over 41,000 Sri Lankan rupees (LKR) and attitude of males regarding their partner attending screening services. The cut off of LKR 41,000 (approximately 400 USD) for an adequate family income was based on the Sri Lankan census department data which defines LKR 41,000 as the average household expenditure per month in 2012/13 [8]. When defining dichotomous variables for comparison, those with primary education and no schooling were taken as one category as basic health education including sexual health is only taught in secondary school upwards.

### Statistical analysis

The findings relevant to descriptive statistics was summarized into proportions and averages (means/SD or percentiles) based on the scales of measurements. Comparisons were evaluated using Mann Whitney *U* test or Chi square tests based on retrospective groupings, variable distributional patterns and scales of measurements. Level of statistical significance was set at *p* < 0.05. Binary logistic regression was used to adjust for confounding factors for statistically significant associations identified in univariate analysis. SPSS statistical software (IBM, United States, Version 18) was used for data analysis.

The ethical clearance for the study was granted by the ethics review committee of Faculty of Medicine, University of Ruhuna, Sri Lanka. All participants were explained the purpose of the study and were required to sign a consent form prior to data collection.

## Results

A total of 282 (n-282) couples were interviewed. The basic demographic characteristics of the sample are summarized in Table 1. The mean age of the male and female partners in the sample was 44 and 43 years respectively. The majority were of Sinhala ethnicity. Approximately, 80 % of the sample had completed at least 11 years of school education. There were few instances of non-consent (less than 15) and in all situations the respondents were too busy to fill the questionnaire.

**Table 1:** Socio-demographic characteristics of the study sample (number of couples - 282)

Variable	Number (%)/Mean ± SD (female partner)	Number (%)/Mean ± SD (male partner)
Mean age	43.05 ± 13.09	44.83 ± 13.82
*Ethnicity (n-282)*		
Sinhala	267 (94.7)	267 (94.7)
Tamil	2 (0.7)	2 (0.7)
Muslim	13 (4.6)	13 (4.6)
*Level of Education (n-280)*		
No schooling	15 (5.3)	10 (3.6)
Primary Education	37 (13.2)	45 (16.1)
Secondary up to O/L examination^b^	43 (15.3)	52 (18.6)
Secondary up to A/L examination^c^	143 (50.9)	134 (47.9)
Tertiary education	43 (15.3)	39 (13.9)
*Employment status (n-254)*		
None or temporary	108 (42.5)	77 (30.3)
Permanent	146 (57.5)	177 (69.7)
Mean family income per month in LKR^a^ (n-241)	54,453 ± 54,968 (Range: 800 – 400,000)	
Mean family savings per month in LKR^a^ (n – 241)	9247 ± 15,498 (Range: 0 – 100,000)	

^a^LKR – Sri Lankan Rupee, 100 LKR = 1 USD (approximately), ^b^O/L – General Certificate of education – Ordinary level (equivalent to 11 years of school education), ^c^A/L - General Certificate of education – Advance level (equivalent to 13 years of school education)

### Awareness of malignancies

As mentioned previously, the awareness of three common female malignancies; breast, cervical and uterine cancers were assessed by a questionnaire with six sections pertaining to risk factors and signs / symptoms of each of the malignancies. After setting a cut off of 50 % as a satisfactory level of awareness, a majority (>50 %) still had an unsatisfactory level of awareness on all three malignancies. These findings are summarized in Table 2.

**Table 2:** Awareness of symptoms and risk factors for female malignancies among participants and their male partners (n-282)

Variable	Numbers with satisfactory knowledge^a^ (%) Female partner	Number with satisfactory knowledge^a^ (%) Male partner
Symptoms and signs of breast cancer	136 (48.2)	116 (41.1)
Risk factors for breast cancer	126 (44.7)	109 (38.7)
Symptoms and signs of cervical cancer	135 (47.9)	122 (43.3)
Risk factors for cervical cancer	103 (36.5)	87 (30.9)
Symptoms and signs of uterine cancer	138 (48.9)	116 (41.1)
Risk factors for uterine cancer	97 (34.4)	78 (27.7)

^a^Satisfactory level of knowledge was defined when a participant identified more than 50 % of correct responses in a test that had both correct and incorrect answers for each of the subsections listed in the table. For purposes of marking, incorrect responses were not given negative marks but response sheets that had marked all responses as either “yes” or “no” were not counted as it may not reflect a genuine effort to answer all questions according to knowledge (five respondents were excluded on this basis)

The level of awareness in females was slightly better than that of males for all three malignancies. More than 50 % of participants (both males and females) did not recognize early initiation of unprotected intercourse or having multiple sexual partners as risk factors for cervical cancer.

### Awareness of screening services and early detection methods for female malignancies

In this regard there was a clear disparity between awareness and utilization of facilities. For example more than 50 % of the sample was aware of Pap smear as a method of screening for cervical cancer but only 7.6 % of the eligible sample (women older than 21 years) had ever undergone one (Table 3). Similarly, 66 % of the total sample were aware of well woman clinics but only 24 % of the eligible sample (women older than 45 years, n-120) had ever attended one. Only 16 % of the eligible sample had attended one within the last 5 years. Even simple measures like regular SBE were not practiced by 60 % of female respondents.

**Table 3:** Awareness and utilization of screening methods, facilities and programmes for early detection of female malignancies

Variable	Female partner, Number (%)	Male partner, Number (%)
Aware of well woman clinics	187 (66.3)	141 (50.0)
Had attended one (n-120)^a^	29 (24.2)	N/A
Had attended one in last 5 years (n −120)^a^	19 (15.8)	N/A
Aware of self-examination of breasts	222 (78.7)	169 (59.9)
Routine practice of self-examination of breasts^b^	108 (38.3)	N/A
Aware of mammography	133 (47.2)	101 (35.8)
Had undergone mammography (n-142)^c^	8 (5.6)	N/A
Aware of pap Smear	144 (51.1)	111 (39.4)
Had undergone pap smear (n-276)^d^	21 (7.6)	N/A

^a^applicable to women over 45 years only,^b^ at least once a year, ^c^applicable to women above 40 years, ^d^Applicable to women over 21 years, N/A-Not applicable

The level of awareness of each screening method/acility was less in males compared to women. Less than half the sample was aware of existence of well woman clinics, Pap smear and mammography. When asked if they had ever actively encouraged their female partner (if eligible) to attend a well woman clinic, only 34 (28.3 %) of males admitted to having suggested that.

### Factors associated with a satisfactory level of knowledge regarding each female malignancy in both sexes

Chi square test was used for univariate analysis of dichotomous outcomes and potential associations. The findings are summarized in Table 4. Statistics for each sex was compared separately. Three factors, namely; having a family income greater than LKR 41,000, having at least a secondary education and having permanent employment all showed a consistent strong association with a better awareness of all three malignancies in both sexes. Having a relative with cancer was associated with a significantly better awareness among males but not in females.

**Table 4:** Univariate analysis of factors associated with satisfactory knowledge of female malignancies, risk ratios (95 % confidence intervals) and *p* values

A satisfactory level of knowledge in following sections	Family income > Rs. 41,000	At least secondary education (female)	At least secondary education (male)	Permanent employment (female)	Permanent employment (male)	Family member with cancer (female)	Family member with cancer (male)
Symptoms and signs of breast cancer	1.84 (1.46 – 2.33), < 0.001	7.63 (2.96–19.72), < 0.001	7.09 (2.73–18.41), < 0.001	1.91 (1.4–2.61), < 0.001	2.34 (1.47–3.71), < 0.001	1.45 (1.1–1.92), 0.027	2.00 (1.56–2.58), < 0.001
Risk factors for breast cancer	1.58 (1.23–2.03), < 0.001	5.6 (2.41–13.01), < 0.001	4.35 (2.01–9.39), < 0.001	2.06 (1.47–2.9), < 0.001	2.78 (1.65–4.67), < 0.001	1.25 (0.89–1.77), 0.265	2.29 (1.77–2.96), < 0.001
Symptoms and signs of cervical cancer	1.71 (1.35–2.16), < 0.001	4.98 (2.32–10.66), < 0.001	5.93 (2.54–13.82), < 0.001	2.31 (1.64–3.26), < 0.001	2.28 (1.46–3.55), < 0.001	1.45 (1.11–1.94), 0.026	1.87 (1.46–2.39), < 0.001
Risk factors for cervical cancer	1.58 (1.17–2.13), < 0.005	23.6 (3.37–165.4), < 0.001	4.15 (1.76–9.76), < 0.001	2.65 (1.69–4.15), < 0.001	2.65 (1.48–4.73), < 0.001	1.38 (0.94–2.05), 0.177	2.84 (2.09–3.85), < 0.001
Symptoms and signs of uterine cancer	1.69 (1.34–2.12), <0.001	5.09 (2.38–10.9), < 0.001	5.62 (2.41–13.13), < 0.001	2.21 (1.57–3.1), < 0.001	2.12 (1.36–3.32), < 0.001	1.28 (0.94–1.73), 0.153	2.00 (1.56–2.58), < 0.001
Risk factors for uterine cancer	1.77 (1.29–2.43), < 0.005	22.2 (3.16–155.8), < 0.001	9.63 (2.44–38.02), < 0.001	2.3 (1.48–3.57), < 0.001	2.18 (1.2–3.92), < 0.005	0.96 (0.58–1.61), 0.88	2.32 (1.62–3.32), < 0.001

Level of significance; *p* < 0.05

### Factors associated with utilization of screening facilities

The findings of univariate analysis for four outcomes (attending well woman clinics, performing SBE, having undergone mammography and pap smear) are summarized in Table 5. Having ever attended a well woman clinic was associated with a satisfactory knowledge of breast, cervical or uterine cancer. Similarly, a better family income, educational status and being permanently employed was also significantly and positively associated with this outcome. Interestingly, the same attributes in the male partner was also significantly associated with the female partner attending well woman clinics. Similar associations were less clear cut for women undergoing mammography or pap smear but the numbers of women that had undergone either investigation were few. Regularly performing SBE was also significantly associated with having a better knowledge of breast cancer in both male and female partners and other demographic factors such as better education in both sexes and better family income. It is noteworthy that active encouragement by men to attend a well woman clinic was significantly associated with better clinic attendance of the female partner as well as her performing regular SBE or undergoing mammography.

**Table 5:** Univariate analysis of factors associated with utilization of health care services by females for early detection of reproductive tract malignancies, risk ratios (95 % confidence intervals) and *p* values

Variable	Attending well woman clinics^a^	Performing self-examination of breasts at least once a year	Having undergone mammography^b^	Having undergone pap smear^c^
*In female partner*				
Satisfactory knowledge on breast cancer	2.21 (1.48–3.3), < 0.001	1.68 (1.32–2.15), < 0.001	1.81 (1.08–3.02), 0.089	NA
Satisfactory knowledge on cervical cancer	3.44 (2.12–5.6), < 0.001	NA	NA	1.42 (0.91–2.19), 0.169
Satisfactory knowledge on uterine cancer	4.01 (2.48–6.49), < 0.001	NA	NA	1.78 (1.22–2.59), 0.02
Family income > Rs. 41,000	2.56 (1.64–4.0), < 0.001	2.34 (1.71–3.19), < 0.001	2.35 (1.54–3.6), 0.011	1.77 (1.21–2.57), 0.02
Permanent employment	2.04 (1.52–2.73), < 0.001	1.9 (1.52–2.37), < 0.001	1.51 (1.02–2.22), 0.174	1.5 (1.15–1.97), 0.04
Having at least a secondary education	1.49 (1.22–1.83), 0.003	1.32 (1.19–1.45), < 0.001	1.43 (1.28–1.6), 0.061	1.19 (1.06–1.33), 0.143
Having a relative with cancer	3.62 (1.32–9.93), 0.015	2.18 (1.15–4.18), 0.021	3.24 (1.13–9.24), 0.072	2.18 (0.94–5.06), 0.087
*In male partner*				
Satisfactory knowledge on breast cancer	4.07 (2.46–6.73), < 0.001	1.74 (1.29–2.34), < 0.001	2.45 (1.43–4.19), 0.02	NA
Satisfactory knowledge on cervical cancer	4.43 (2.57–7.64), < 0.001	NA	NA	1.47 (0.91–2.39), 0.157
Satisfactory knowledge on uterine cancer	4.39 (2.37–8.11), < 0.001	NA	NA	0.92 (0.45–1.87), 1.000
Permanent employment	1.44 (1.13–1.83), 0.015	1.63 (1.37–1.93), < 0.001	1.37 (1.05–1.78), 0.273	1.38 (1.13–1.69), 0.03
Having at least a secondary education	1.52 (1.26–1.84), 0.001	1.29 (1.16–1.43), < 0.001	1.45 (1.29–1.62), 0.059	1.21 (1.08–1.35), 0.07
Having a relative with cancer	5.75 (2.53–13.09), < 0.001	1.37 (0.83–2.27), 0.262	2.15 (0.79–5.89), 0.170	1.42 (0.63–3.2), 0.378
Awareness of well woman clinics	2.6 (1.89–3.59), < 0.001	1.72 (1.37–2.16), < 0.001	1.92 (1.43–2.57), 0.016	1.25 (0.87–1.78), 0.28
Awareness of Pap smears	2.83 (1.85–4.33), < 0.001	NA	NA	1.22 (0.76–1.96), 0.435
Awareness of self-examination of breasts	2.15 (1.64–2.8), < 0.001	1.56 (1.31–1.85), < 0.001	NA	NA
Awareness of mammography	3.93 (2.29–6.72), <0.001	NA	2.91 (1.86–4.56), 0.003	NA
Active encouragement of partner to attend a well woman clinic^a^	5.01 (2.87–8.73), < 0.001	1.72 (1.24–2.37), 0.001	2.78 (1.79–4.33), 0.004	1.62 (1.03–2.53), 0.091

^a^Only for women over 45 years, ^b^ for women over 40 years only, ^c^For women over 21 years only, N/A – not applicable, Level of significance; *p* < 0.05

Binary logistic regression was carried out independently for following outcomes; a) attending a well woman clinic, b) performing regular SBE and c) having ever undergone Pap smear. The independent variables assessed for each analysis were a) having at least a secondary education (for both sexes separately), b) having permanent employment (for both sexes separately) and c) having a family income above LKR 41,000 per month. For having attended a well woman clinic, only permanent employment status of women remained significant (p = 0.038). For having a Pap smear none of the variables remained significant and for performing SBE, following were significant associations in the final output; at least having a secondary education in females (p = 0.027), permanent employment status of males (p = 0.022) and having a family income above LKR 41,000 (p = 0.045).

## Discussion

The most noteworthy observation in this study is that the awareness of all three common female malignancies was unsatisfactory in the sample for both sexes. Understandably, the awareness of females was slightly better than that of males. This is not surprising given the fact that there is no formal communal education on any of these malignancies. Even the school curricula do not address the signs and symptoms of these female malignancies. Even if the topics were to be covered in school, the target group would be an age group least affected by any of these malignancies. Well woman clinics do educate women on these malignancies but only women above 45 years of age are eligible to attend these clinics (service is not available to men). In short, there is no formal community-wide educational programme targeting women in sexually active reproductive age groups. The education and awareness on these malignancies are sporadically picked up by the general public from newspapers, magazines, internet and televised healthcare programmes. These are by no means a substitute for an organized all inclusive centrally coordinated community based health awareness programme.

Previous studies, both local and international have noted the poor level of awareness of women with regard to these malignancies. A school based study in the metropolitan Colombo district of Sri Lanka (in which the national capital is located), showed that awareness of common signs and symptoms of breast cancer to be grossly inadequate [2]. One third of the sample did not have any satisfactory knowledge on signs, symptoms, screening and treatment modalities of breast cancer. Only 36 % of the sample had heard about mammography. Similarly, another study on awareness of pap smear as a screening method for cervical cancer showed that out of 188 women interviewed, only 111 (59 %) were aware of pap smear and only 34 (18.1 %)had ever undergone screening [4]. Again this study was conducted in the capital, Colombo. The hypotheses raised in both studies were that level of awareness can be even lower in more peripheral and rural areas of Sri Lanka. That assumption appears to be correct at least when considering the Galle district in the Southern province as the percentage uptake of Pap smear was even lower in our data.

There are several international studies which have assessed certain aspects studied in this survey. A cross sectional survey in Qatar of 1063 participants showed that only 29 and 25 % of women (aged 35 or older) knew about SBE and mammography, respectively [9]. Only 14 % admitted to practicing SBE monthly and only quarter of the eligible population has had regular mammography. A study in Lagos, Nigeria recorded that out of 218 women screened, 156 (72 %) were aware of breast cancer but the sample bias was obvious with the entire sample coming from a breast cancer treatment clinic [10]. The awareness is also better in certain groups of women such as healthcare workers. However, evidence suggests that even in healthcare workers, awareness doesn’t convert well in to practice. A survey of nursing students in Turkey showed that 81 % of the sample had a satisfactory level of awareness of breast cancer due to their training but only 64 % of the sample practiced regular SBE [11]. There are numerous other studies from low and middle income countries showing unsatisfactory level of awareness of these key malignancies and low uptake of screening practices [12, 13]. The surveys from high income countries report better numbers with regard to awareness [14, 15], but findings differ depending on different socioeconomic and occupational groups interviewed [16]. On the topic of cervical cancer, studies have noted similar deficiencies [17]. A review found that compared to high income countries the effect of pap smear in reducing cervical cancer related mortality is less in low and middle income countries due to poor coordination of screening, unsatisfactory public participation, limited access to healthcare and lack of quality assurance [18].

It is notable that in both females and their male partners, better awareness of all three malignancies showed a consistent strong association with having a better education, family income and permanent employment in univariate analysis. Socio-economic stability may increase the opportunity for better education and proper health seeking behaviour. It also increases the buying power to access media and internet plus formal health educational programmes. They are also more likely to have health insurance schemes supported by employers that fund routine check-ups and screening. Having a relative affected by cancer showed a strong association with better awareness in males but not in females. This is probably because women are more likely to be aware of these malignancies regardless of having an experience of a relative having cancer. However males are more likely to be knowledgeable about this group of malignancies that does not affect them when a relative falls victim to a similar illness.

Despite scarcity of data from Sri Lanka, there is evidence from other countries that better socio-economic standing and better education is associated better awareness of malignancies considered. The previously mentioned study in Qatar noted that better education (secondary or tertiary) was strongly and significantly associated with better awareness of breast cancer (*p* < 0.001) [9]. However being employed did not confer such an advantage. Still, it is observed that unlike our sample where a majority of women were employed, in this study, almost two third of the sample were unemployed. Income disparity and socioeconomic standing at a national level is shown to be related to successful utilization of screening services for cervical cancer with countries with better socioeconomic standing having better organized screening facilities and more detection rates (which is partly dependent on customers being knowledgeable and accessing these services) [18]. It is also noted that all female malignancies are increasing in low and middle income countries compared to high income countries [19]. Unlike the more popular breast and cervical cancers, studies on awareness of endometrial cancer are limited. From the data available, even in high income countries, the knowledge on this type of cancer is considered to be unsatisfactory [20, 21].

Awareness does not always convert in same numbers to practice. In Sri Lanka, it has been demonstrated previously that most people who are aware of screening procedures for breast cancer do not undergo screening [2]. Agreeing with that observation, in this sample also, the overall numbers of women actually attending well woman clinics, undergoing mammography or Pap smear were small. Nevertheless, there was a significant positive association of being aware of malignancies and attending well woman clinics. Whether attendance improved knowledge or vice versa is difficult to establish from this study. In addition, better socio-economic standing, better educational background, better awareness of female malignancies by male partner and active encouragement by male partner was also significantly and positively associated with female partner attending well woman clinics or performing SBE. It is a plausible hypothesis that better awareness by males will eventually lead to better female participation in screening services. Males can play an active role in coaxing women to participate in screening programmes especially in countries like Sri Lanka where subtle male dominant gender norms are prevalent.

Despite being considered as independent variables for univariate analysis; educational level, employment status and family income are not mutually exclusive. Obviously a better income leads to better employment and educational opportunities and vice versa. Considering these confounding effects we carried out a binary logistic regression of all variables that were significantly associated with better utilization of screening services in the univariate analysis. While the results varied according to the screening service studied, for attending well woman clinics, only permanent employment status of women remained significant. This highlights the importance of female empowerment, especially by financial means that may lead to positive health seeking behaviours. While there were some significant associations in the final output for other screening services studied (mammography, SBE) numbers utilizing these services were few to make any valid conclusions.

The traditional approach of only targeting women in health educational programmes on female malignancies needs to change. In Ghana, it has been shown that as a result of female targeted awareness programmes, the level of knowledge on breast cancer among men was unsatisfactory. However, when educated on the problem, they were willing to encourage female partners to attend screening services or perform SBE [22]. Similarly a qualitative study with focus group discussions in Mexican immigrants in United States showed less awareness about breast and cervical cancer among men compared to females. However, males had very positive attitudes towards screening once educated on the topic [23]. The education of males is even more important when it comes to cervical cancer as now a vaccine is available against Human Papilloma Virus (HPV). Fathers, husbands and male partners being educated on the topic can increase the vaccine uptake in target adolescent female population. In addition, the vaccine also protects against HPV induced cancers in males. A study in Japan showed that knowledge about HPV vaccine among fathers to be poor (small sample size, n −27) but once educated, the acceptability for the vaccine was high [24]. These observations suggest that health education on female malignancies for both sexes is a better way forward in reducing cancer related morbidity and mortality.

### Limitations

This study was limited to one district of the Southern province of Sri Lanka and hence cannot draw conclusions on other regions of the country. However, it draws on the observations of similar studies conducted in the Colombo district of Western province which is a comparatively more populated, metropolitan area to identify similarities and dissimilarities. A satisfactory awareness of female malignancies was arbitrarily decided at a cut off of 50 % under each section. Equal weightage was given to each sign/symptom or risk factor. However, awareness of some risk factors or symptoms is more important than others when it comes to prevention (modifiable vs. non-modifiable risk factors). The results of the logistic regression are difficult to interpret due to the collinearity of the independent variables. For example though considered as “independent” variables, the individuals with permanent employment also had a higher family income. Similarly those with a better level of education were more likely to be employed and hence to have a higher family income.

## Conclusions

This cross sectional survey of women of reproductive and post-menopausal age groups and their male partners in Southern Sri Lanka showed that level of awareness of breast, cervical and endometrial carcinoma to be unsatisfactory in both sexes. Overall, males had a lower level of awareness compared to women. Better socioeconomic standing, being permanently employed and having a higher family income were positively and significantly associated with better awareness of malignancies for both sexes. Some of these variables remained significantly associated with better utilization of certain screening tests by women in both univariate analysis and in binary logistic regression.

Female malignancies are on the rise in low and middle income countries and several studies including those in Sri Lanka have shown that the level of awareness of these malignancies to be unsatisfactory among women. This study showed that awareness is comparatively worse among men. Furthermore, studies including this have derived evidence in support of educating and harnessing support of male partners to increase the uptake of screening services by women. Educating males is also important as breast cancer can also affect men and the availability of HPV vaccine prevents HPV induced malignancies in both sexes. There should be an organized community based healthcare and educational programme that invites participants from both sexes and it is recommended to have an alternative other than well woman clinics (which currently targets women over 45 years of age) for this purpose. It is also important to start health education in early adulthood as several screening methods such as SBE and Pap smear are relevant to women from this age group onwards.

## Abbreviations

MOH division: Medical Officer of Health division
WWC: Well Woman Clinic
Pap Smear: Papanicolaou smear
SBE: Self breast examination
LKR: Sri Lankan Rupee
HPV: Human Papilloma Virus
